# Extracellular Matrix Stiffness: New Areas Affecting Cell Metabolism

**DOI:** 10.3389/fonc.2021.631991

**Published:** 2021-02-24

**Authors:** Heming Ge, Mengxiang Tian, Qian Pei, Fengbo Tan, Haiping Pei

**Affiliations:** Department of General Surgery, Xiangya Hospital, Central South University, Changsha, China

**Keywords:** extracellular matrix stiffness, metabolic reprogramming, glucose metabolism, lipid metabolism, amino acid metabolism

## Abstract

In recent years, in-depth studies have shown that extracellular matrix stiffness plays an important role in cell growth, proliferation, migration, immunity, malignant transformation, and apoptosis. Most of these processes entail metabolic reprogramming of cells. However, the exact mechanism through which extracellular matrix stiffness leads to metabolic reprogramming remains unclear. Insights regarding the relationship between extracellular matrix stiffness and metabolism could help unravel novel therapeutic targets and guide development of clinical approaches against a myriad of diseases. This review provides an overview of different pathways of extracellular matrix stiffness involved in regulating glucose, lipid and amino acid metabolism.

## Introduction

The extracellular matrix (ECM), mainly composed of collagen, fibronectin, laminin, elastin, and thrombospondin, is a non-cellular component of the cellular microenvironment (1, 2). During embryonic development and tumorigenesis, there is an increase in deposition and cross-linking of collagen as well as hyaluronan acid content, resulting in increase of ECM’s stiffness (3–5). Existing evidence has also implicated ECM stiffness in tumor development (6, 7).

Metabolism provides energy and biomass for cellular activity and proliferation (8), with normal cells meeting their metabolic needs primarily by regulating glucose, lipid and amino acid metabolism. In cancer cases, metabolic reprogramming is a common phenomenon that allows cancer cells to proliferate, survive, and spread in an altered microenvironment. The enhanced metabolism of glucose, lipid, and amino acid in cancer cells provides energy, membrane lipid molecules, signaling molecules, and nucleotides for rapid proliferation (9–11). Previous studies have described the role played by ECM stiffness in regulating the cellular metabolic reprogramming, especially in tumors. Specifically, it has been suggested that both *in vivo* and *in vitro* experimental changes in tumor matrix stiffness or stiffness-related metabolic reprogramming can significantly inhibit tumor growth and invasive metastasis. Therefore, targeting metabolic reprogramming associated with tumor stiffness could be a potential therapeutic strategy for clinical treatment of cancer.

In this review, we briefly introduce the stiffness composition of ECM and the mechanobiological coupling pathway. Furthermore, we comprehensively reviewed regulation of ECM stiffness on glucose, lipid and amino acid metabolism, as well as the associated effects on cell proliferation, survival, and invasion. Overall, we provide new ideas for understanding cellular metabolism, which are expected to guide development of new strategies for the treatment of tumors.

## Extracellular Matrix Components and Mechanical Biological Coupling Pathway

ECM comprises several components. Among them, collagen and hyaluronan acid are the main ones that affect its stiffness. Previous studies have shown that increase in deposition and cross-linking of collagen as well as hyaluronan acid contents increase ECM’s stiffness (3–5, 12). In addition, this stiffness can transfer physical signals from the ECM to intracellular matrix through mechanical conduction, thereby change the biological behavior of the cell.

Studies have also shown that both collagen receptor integrin and hyaluronan acid receptor CD44 are involved in the mechanical conduction of ECM stiffness (13). Specifically, hyaluronan acid with different molecular weights binds to its receptor CD44, whereas collagen components of various subtypes bind to corresponding integrin receptor subtypes, regulating downstream signal pathways and producing different biological effects (**Table 1**).

**Table 1:** ECM components and mechanical biological coupling pathway.

ECM components	Receptor protein	Signal pathway	Cell model	Animal model	References
Collagen	β1 integrin	integrin-FAK-PI3K-Akt	Ha-ras human MCF10AT MECs	NOD/SCID mice	Matrix Crosslinking Forces Tumor Progression by Enhancing Integrin Signaling (3)
Collagen type I	β1 integrin	integrin–N-cadherin	astrocytes	female C57BL/6J mice	Interaction of reactive astrocytes with type I collagen induces astrocytic scar formation through the integrin– N-cadherin pathway after spinal cord injury (14)
Collagen type I	β1 integrin	integrin-FAK-YAP	Repairing Epithelium	C57BL/6J mice	YAP/TAZ-Dependent Reprogramming of Colonic Epithelium Links ECM Remodeling to Tissue Regeneration (15)
Collagen type I	CD44	CD44-RhoA-YAP	fibroblasts	Male C57BL/6 mice	Targeting Mechanics-Induced Fibroblast Activation through CD44-RhoA-YAP Pathway Ameliorates Crystalline Silica-Induced Silicosis (16)
Collagen type V	αvβ3 and αvβ5 integrins	NA	cardiac fibroblast	Murine models	Type V Collagen in Scar Tissue Regulates the Size of Scar after Heart Injury (13)
HA	CD44	CD44-ERM-actin cytoskeleton	human kidney cell line 293	NA	CD44 is required for two consecutive steps in HGF/c-Met signaling (17)
HA	CD44	CD44-AFAP-110-actin cytoskeleton	MDA-MB-231 cells	NA	Interaction of Low Molecular Weight Hyaluronan with CD44 and Toll-Like Receptors Promotes the Actin Filament-Associated Protein 110-Actin Binding and MyD88-NFjB Signaling Leading to Proinflammatory Cytokine/Chemokine Production and Breast Tumor Invasion (18)
HA	CD44	CD44-Rho GTPases	hippocampal neurons	Wistar rats postnatal day 0	CD44 - a novel synaptic cell adhesion molecule regulating structural and functional plasticity of dendritic spines (19)
HA	CD44	CD44-ERM-cytoskeleton	macrophage	female C57Bl/6 mice	Transmembrane pickets connect cyto-and pericellular-skeletons forming barriers to receptor engagement (20)
HA	NA	RTK-ZFP36-TXNIP/ MYC-TXNIP	LiSa-2 liposarcoma cells	White SCID (Beige) mice	Extracellular Matrix Remodeling Regulates Glucose Metabolism through TXNIP Destabilization (21)
NA	β1 integrin	integrin-GSK3β-β Catenin	Huh7 cells	NA	Higher Matrix Stiffness Upregulates Osteopontin Expression in Hepatocellular Carcinoma Cells Mediated by Integrin β1/GSK3β/β-Catenin Signaling Pathway (22)
NA	E cadherin	E cadherin-LKB1-AMPK	MCF10A/MDCK II	NA	Linking E-cadherin mechanotransduction to cell metabolism through force mediated activation of AMPK (23)

In different cells or animal models, extracellular matrix components combine with their receptors and affect different downstream signaling pathways. HA, hyaluronic acid. NA, not available.

This affirms the need to analyze composition and proportions of ECM across different tissue samples during clinical or basic translational studies. In addition, targeted anti-downstream signaling pathways or receptors of different ECM components and compositions can achieve precise and individualized therapeutic effects in the extracellular microenvironment.

## ECM Stiffness and Glucose Metabolism

**Figure 1:**
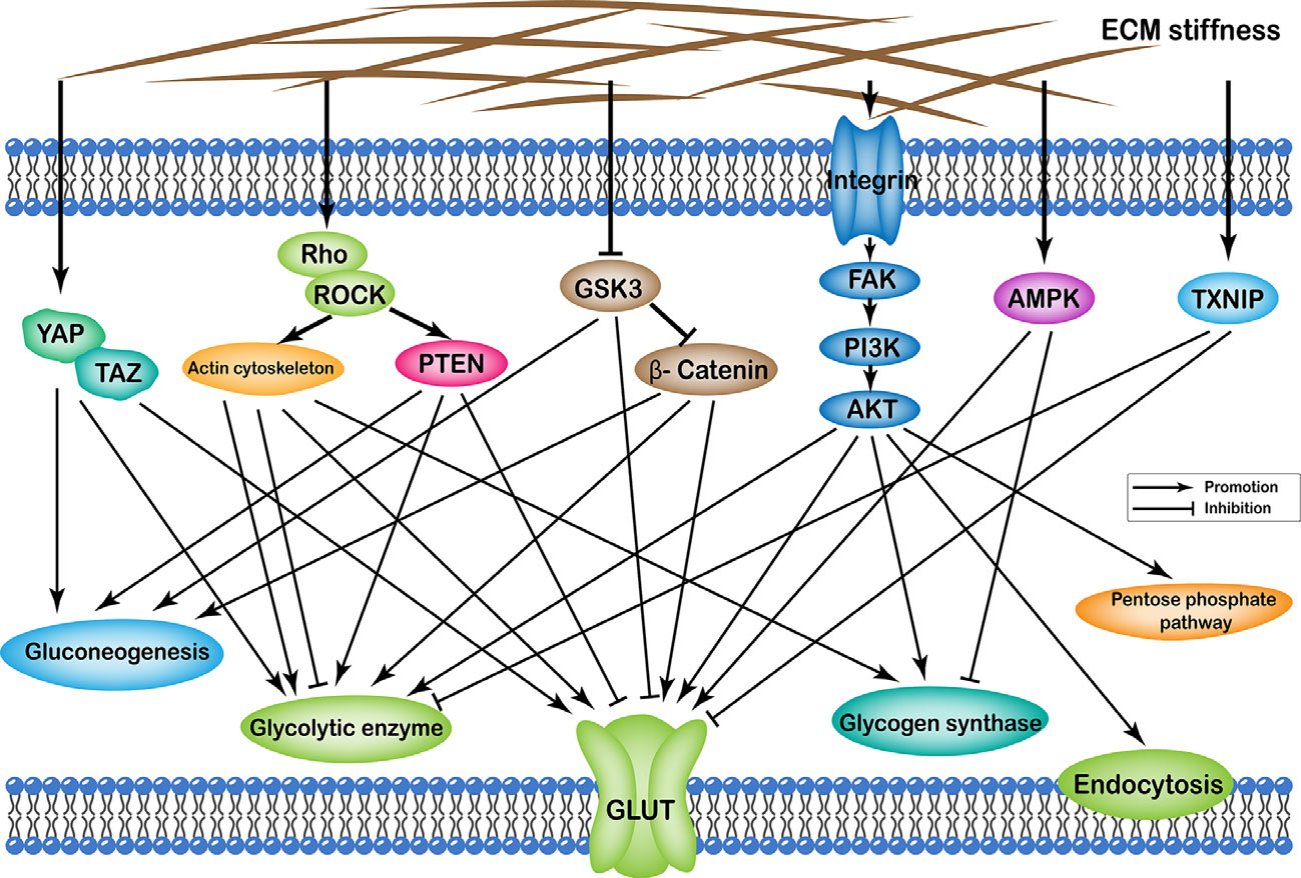
Profiles of pathways through which extracellular matrix stiffness affects glucose metabolism. Extracellular matrix stiffness affects glucose metabolism in the following seven pathways: (i) YAP/TAZ pathway; (ii) TXNIP pathway; (iii) Rho/Rock-actin cytoskeleton pathway; (iv) Rho/Rock-PTEN pathway; (v) integrin-FAK-PI3K-Akt pathway; (vi) GSK3 pathway; and (vii) AMPK pathway. The effects of extracellular matrix stiffness on cellular glucose metabolism can ultimately be summarized as follows: (i) Changes in the number of glucose transport proteins in the cell membrane; (ii) Alteration of glycolytic enzyme activity; (iii) Alteration of glycogen synthase activity; (iv) Expression of gluconeogenic genes; (v) Enhancement of the pentose phosphate pathway; and (vi) Endocytosis of cells.

Effects of ECM stiffness on cellular glucose metabolism are mediated by multiple pathways, which are broadly categorized as: (i) YAP/TAZ pathway; (ii) TXNIP pathway; (iii) Rho/Rock-actin cytoskeleton pathway; (iv) Rho/Rock-PTEN pathway; (v) integrin-FAK-PI3K-Akt pathway; (vi) GSK3 pathway; and (vii) AMPK pathway. Furthermore, the effects of passageways regulated by ECM stiffness on cellular glucose metabolism can ultimately be summarized as follows: (i) changes in the number of glucose transport proteins in the cell membrane; (ii) alteration of glycolytic enzyme activity; (iii) alteration of glycogen synthase activity; (iv) expression of gluconeogenic genes; (v) enhancement of the pentose phosphate pathway; and (vi) cell endocytosis (**Figure 1**).

YAP/TAZ is a key molecule in the mechanobiological coupling signaling pathway, owing to its involvement in embryo and tumor development where it promotes cell proliferation and survival. The mechanisms through which ECM stiffness upregulates YAP/TAZ are diverse, and may be regulated differently across different cell types. Generally, these mechanisms are broadly categorized as: (i) effect on cytoskeletal tension (24); (ii) effect on hyaluronic acid (HA) receptor CD44 (16); (iii) activation on the MAPK signaling cascade in hepatic cancer cells (25); (iv) ECM stiffness can affect cellular metabolism through integrin (discussed in a later section), and it has been reported that integrin affects cellular metabolism through YAP/TAZ (26). However, it is unclear whether ECM stiffness can activate YAP/TAZ through integrin. (v) ECM stiffness regulates AMPK (23, 27), which is also involved in regulating YAP/TAZ signals (28). Therefore, we hypothesized that ECM stiffness could regulate metabolism through AMPK-mediated YAP/TAZ signals.

Previous studies have also shown that stiffness upregulates YAP/TAZ expression in various types of cells (24, 25, 29–32), such as hepatocellular carcinoma (25), hepatic stellate cells (33) and pulmonary artery endothelial cells (30). Consequently, this YAP/TAZ-mediated upregulation promotes uptake and utilization of cellular glucose, increases glycolysis, and influences glycogenolysis. The activated YAP/TAZ can be involved in glucose metabolism in three general ways. Firstly, it increases expression of glucose transport proteins. For example, The YAP-TEAD was shown to directly regulate GLUT1 (25, 34) and GLUT3 (26, 28) transcription, thereby promoting cellular uptake of glucose, which supplies more energy to cells and is also involved in nucleotide biosynthesis. In zebrafish, WZB117-mediated inhibition of GLUT1 and mutations in YAP were both shown to reduce glucose uptake and subsequent nucleotide synthesis leading to reduced liver volume (34). Thus, it is possible that matrix stiffness is involved in regulating liver growth and size by influencing YAP/TAZ-mediated glucose uptake. Secondly, activated YAP/TAZ affects glucose metabolism by increasing expression of key glycolytic enzymes, such as hexokinase 2 (HK2) (25, 35–37), lactate dehydrogenase A (LDHA) (25, 30, 32), pyruvate kinase M2 (PKM2) (38), and 6-phosphofructo-2-kinase/fructose-2,6-biphosphatase 3 (PFKFB3) (37). This consequently promotes glycolysis, thereby providing more energy for cellular activities and more carbon skeletons to the cell. Knocking out of the YAP gene in human hepatocellular carcinoma (HCC) cell lines, HepG2, and MHCC97L cells, cultured in the stiff ECM, resulted in downregulation of glycolytic enzymes HK2 and LDHA, which subsequently reduced the migration capacity of cancer cells (25). Subsequently, HCC cells cultured on stiff hydrogels with HK2 downregulated by siRNA knockdown, exhibited impaired ability to migrate compared to control cells. Moreover, there were no significant differences in cell migration when the cells were incubated on hydrogels of different stiffness after HK2 knockdown (25). Knocking down of HK2, a gene downstream of YAP, in breast cancer cells MCF7 also inhibited the migration ability of the cells (35). On the other hand, Zheng et al. found that the induced expression of HK2 and PFKFB3 by YAP overexpression in a subcutaneous breast cancer xenograft model in nude mice increased tumor weight and tumor size (37). In a subcutaneous nasopharyngeal carcinoma model of nude mice, 3-BrPA-mediated inhibition of HK2, downstream of YAP/TAZ, also significantly inhibited the growth of nasopharyngeal carcinoma in mice (36). Thirdly, upregulated YAP/TAZ can affect key enzymes of the gluconeogenesis, hence affecting glucose metabolism. Functionally, YAP/TAZ represses expression of phosphoenolpyruvate carboxykinase 1 (39) and glucose-6-phosphatase catalytic subunit (39) by inhibiting the ability of PGC1α to bind to and activate transcription of the promoters of gluconeogenic genes. Furthermore, YAP/TAZ has also been shown to upregulate expression of pyruvate carboxylase (30). In summary, ECM stiffness regulates glucose uptake, utilization, and gluconeogenesis *via* the YAP/TAZ signaling pathway both *in vitro* and *in vivo*. This regulation subsequently affects growth, apoptosis, and migration of cancer cells. Reducing ECM stiffness or inhibiting the YAP/TAZ signaling pathway through different ways may delay tumor progression.

ECM stiffness has been shown to regulate glucose metabolism by influencing thioredoxin-interacting protein (TXNIP), a negative moderator of cellular glucose uptake. For instance, Sullivan et al. found that decreasing hyaluronic acid (HA), the main component of ECM stiffness, decreases intracellular TXNIP (21). Short-term reductions in HA content activate RTK signals and promote ZFP36 expression, which results in post-transcriptional regulation and a decrease in TXNIP. Conversely, long-term reductions in HA content increase MYC signals and inhibit TXNIP transcription (21, 40–42).

Effects of TXNIP on glucose metabolism can be categorized as follows; Firstly, TXNIP affects glucose uptake by regulating glucose transport proteins. Consequently, this reduces GLUT1 at the plasma membrane by inhibiting GLUT1 transcription (21, 43) and promoting internalization of GLUT1 and GLUT4 at the plasma membrane (21, 43, 44). Overexpressing TXNIP in human prostate cancer PC3 cells reduced glucose uptake as well as ATP levels (42). A study on triple-negative breast cancer reported that TXNIP overexpression resulted in reduced glucose uptake, impaired cell proliferation, and elevated apoptosis. These findings indicate that its expression is associated with reduced overall survival and reduced metastasis-free survival (40). Secondly, TXNIP inhibits glycolysis and subsequent biological effects (41, 42, 45). For example, its knockdown in breast cancer cells resulted in increased cellular glycolysis and the speed of cell migration (21). In another study, Ji et al. found that TXNIP mediated a reduction in proliferation of cultured human pancreatic cancer cell lines, such as PANC-1 and SW1990 cells, and inhibited the colony-forming ability of pancreatic ductal adenocarcinoma (PDAC) cells (41). In addition, overexpressing TXNIP was found to inhibit progression of the cell cycle and eventually arrest it at the G 2-M phase, whereas its downregulation predicted poor prognosis for PDAC. Therefore, ECM stiffness can regulate cell proliferation and migration through action of TXNIP in cell glucose uptake and glycolysis, and affecting the prognosis of tumor patients.

ECM stiffness has also been shown to affect the actin cytoskeleton by activating Rho/ROCK signaling, which in turn regulates cellular metabolism (5, 16, 46–48). Several ways through which the actin cytoskeleton is involved in the regulation of glucose metabolism have been reported. ECM stiffness can influence glucose uptake by regulating translocation of the glucose transport protein GLUT4 to the cytomembrane (49–51). Besides, most glycolytic enzymes, except hexokinase that bind to mitochondria, are thought to bind to the cytoskeleton, which regulates activity of glycolytic enzymes (52). Park et al. demonstrated that the actin cytoskeleton regulates glycolytic enzyme phosphofructo kinase (PFK) by limiting migration and radius of action of the E3 ubiquitin ligase tripartite motif (TRIM)-containing protein 21 (TRIM21) (48). Functionally, TRIM21 enhances PFK degradation by promoting the role of proteasome (48, 50, 53, 54). In fact, PFK downregulation and effective of glycolysis were observed in human bronchial epithelial cells following blebbistatin-mediated inhibition of myosin II (48). Huang et al. found that ECM stiffness decreased the glycolysis of human colon carcinoma cell line HCT-116 by increasing the density of actin filament and making aldolase combine with actin cytoskeleton in an inactive form (53, 55, 56). In addition, the actin cytoskeleton may regulate glycolysis by affecting glycogen synthesis by regulating intracellular translocation of glycogen synthase (50). Therefore, ECM stiffness plays a key role in regulating cellular glucose uptake, glycolysis and glycogen synthesis by affecting Rho/Rock-actin cytoskeleton. Overall, these processes balance intracellular energy homeostasis and provide energy for cellular life activities.

Apart from regulating cell metabolism, through Rho/Rock-actin cytoskeleton, ECM stiffness can also regulate cell metabolism *via* the Rho/Rock-non-actin cytoskeleton pathway, such as Rho/Rock-PTEN. For example, Li et al. found that RhoA/ROCK can mediate PTEN phosphorylation and activation in leukocytes and human transfected embryonic kidney cells (57). Additionally, activated PTEN up-regulates expression of glycolytic enzymes HK2 and PKM2 (58), thereby increasing the expression of gluconeogenic genes G6Pase and PEPCK (59). However, activated PTEN has also been shown to reduce the amount of GLUT1 on the cell membrane and lower glucose uptake into the cell (60).

Integrin is a family of cell-surface receptors that translate mechanical signals from the ECM into molecular biological signals within the cell. Previous studies have shown that ECM stiffness can stimulate FAK activation by enhancing integrin signaling, thereby activating PI3K/Akt signaling (3, 61–64). Activated Akt signaling regulates cellular glucose metabolism through multiple pathways, and increases the amount of glucose transport proteins in the cell membrane. For example, *in vitro* experiments revealed that Akt could stimulate expression of GLUT1 (65, 66) and GLUT3 (67). However, in a hematopoietic cell line FL5.12, activated Akt, without increasing GLUT1 synthesis, promoted the translocation of GLUT1 to the cell surface (68). In addition, activated Akt was also found to stimulate translocation of GLUT4 to the cell membrane in different types of cells (65, 67, 69, 70). Knocking down of the integrin or GLUT3 gene significantly delayed the *in situ* growth of glioblastoma in immunosuppressed mice orthotopic transplantation model (26). Furthermore, activated Akt increases the activity of glycolytic enzymes. For example, Akt was found to increase cellular HK activity (68, 71), preventing degradation of phosphofructose kinase (PFK), as well as maintaining and activating PFK activity (68). In Rat1a cells, hexokinase, a hexose kinase downstream of Akt, was successfully inhibited cytochrome c release and led to apoptosis (71). Besides, activated Akt has been shown to maintain the pentose phosphate pathway (68), as well as cause an increase in glycogen synthase activity and promote glycogen synthesis (72). Lastly, activated Akt has been shown to increase cellular endocytosis thereby promoting glucose transport (72). Taken together, these findings suggest that ECM stiffness is involved in regulating survival, apoptosis, and tumorigenic capacity of cancer cells by increasing the number of glucose transporters on the cell membrane, improving activity of glycolytic enzyme, as well as enhancing the pentose phosphate pathway and glycogen synthesis through integrin and its downstream signaling pathways.

Previous studies have also shown that ECM stiffness can inactivate phosphorylated GSK3 through integrin-mediated phosphorylation of glycogen synthase kinase-3 (GSK3) (22). Specifically, GSK3 downregulates GLUT4 (73) and upregulates gluconeogenic gene glucose-6-phosphatase (G6Pase) expressions (74), as well as phosphoenolpyruvate carboxykinase(PEPCK) transcription by phosphorylating the cAMP-responsive element transcription factor (73, 74). This in turn regulates glucose homeostasis. In addition, GSK3 has been shown to phosphorylate and degrade *β*-catenin *via* the proteasome (75–78), thereby suppressing cellular glycolysis. *β*-Catenin increases the amount of GLUT1 (79, 80) and GLUT4 (81) in the cell membrane, and also promotes expression of glycolytic enzymes HK2 (79, 80), PKM2 (79, 80), LDHA (79, 80), and LDHB (81). Apart from this, *β*-Catenin has also been implicated in upregulating expression of cellular gluconeozymes G6Pase (82), PEPCK (82), and pyruvate carboxylase (83). Overall, these findings indicate that ECM stiffness, through GSK3 and *β*-catenin, play a key role in regulating glucose transporter, gluconeogenic gene expression and glycolytic enzyme activity, thereby affecting glucose metabolism.

ECM stiffness has been reported to activate the AMPK (23, 27), an essential metabolic regulator that, once activated, shuts down energy-consuming anabolic processes and activates catabolic pathways to produce energy and achieve an energy steady state in a cell. ECM stiffness’s involvement in metabolism is primarily related to ATP production through activation of AMPK. Specifically, activated AMPK increases GLUT4 expression (84, 85) and displacement to the membrane *via* the AMPK-p38 MAPK signaling pathway (84, 86), thereby increasing glucose uptake.

In addition, activated AMPK can also phosphorylate and inactivate glycogen synthase site 2 (87), thus reducing glycogen synthesis energy consumption during glycogen synthesis. Apart from increasing ATP production from glucose metabolism, activation of AMPK by matrix stiffness also increases fatty acid *β*-oxidation to generate ATP. Therefore, ECM stiffness reduces energy consumption and increases ATP production through AMPK signaling, which provides energy for the actin cytoskeleton deformation to resist extracellular forces (23).

## ECM Stiffness and Lipid Metabolism

**Figure 2:**
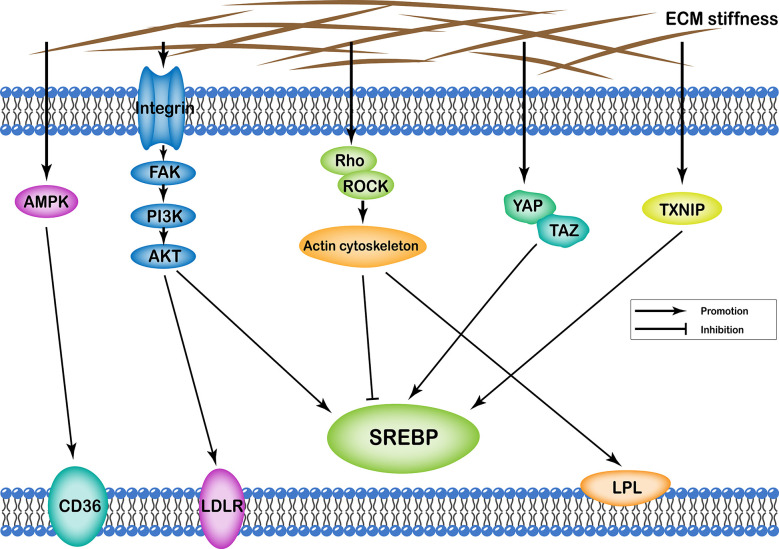
Profiles of pathways through which extracellular matrix stiffness affects lipid metabolism. Extracellular matrix stiffness affects lipid metabolism in the following five pathways: (i) integrin-FAK-PI3K-Akt pathway; (ii) YAP/TAZ pathway; (iii) AMPK pathway; (iv) Rho/Rock-actin cytoskeleton pathway; and (v) TXNIP pathway. The effects of extracellular matrix stiffness on cellular lipid metabolism can be described as upregulation of SREBP, LDLR, LPL and CD36.

ECM stiffness can affect cellular lipid metabolism through several different pathways, including (i) integrin-FAK-PI3K-Akt; (ii) YAP/TAZ; (iii) AMPK; (iv) Rho/Rock-actin cytoskeleton; (v) Rho/Rock-PTEN; and (vi) TXNIP. Transcription of various enzymes involved in cellular lipid metabolism is mainly regulated by the sterol regulatory element-binding protein (SREBP). Particularly, SREBP1 and SREBP2 regulate synthesis of cellular fatty acids and cholesterol, respectively. In addition, ECM stiffness regulates synthesis of various enzymes involved in lipid metabolism through different pathways that regulate both SREBP1 and SREBP2. Apart from this, ECM stiffness further participates in cell lipid metabolism by regulating low-density lipoprotein receptor (LDLR), lipoprotein lipase (LPL), and fatty acid transporter CD36 (**Figure 2**).

ECM stiffness causes FAK activation due to enhanced integrin signaling, which subsequently activates PI3K/Akt signaling. Activated Akt signaling has further been implicated in lipid metabolism in two ways; Firstly, Akt can regulate SREBP1 by activating CRTC2, which further enhances SREBP’s activity by inhibiting degradation of SREBP1 and SREBP2 (88–94). Activated SREBP upregulates fatty acid synthase (FAS) (89–91, 93), Stearoyl-CoA desaturase 1 (SCD-1) (89, 91), ATP-citrate lyase (ACL) (90), and Acetyl-CoA carboxylase (ACC) (90), thereby promoting fatty acids and triglycerides biosynthesis in cells. Furthermore, SREBP upregulates HMG-CoA synthase (93), a key ketogenesis factor. Secondly, Akt signaling has been shown to promote cellular uptake of cholesterol by upregulating LDLR (90, 95). Overexpressing SREBP1 as well as FAS, ACC, ACL, and SCD-1 downstream of AKT signaling in HCC cell lines was found to accelerate growth of cancer cell and arrest apoptosis. Conversely, siRNA-mediated silencing of the above five genes inhibited growth of cancer cell and elevated apoptosis (92).

ECM stiffness has also been shown to upregulate YAP/TAZ, which subsequently regulates SREBP expression (33). This affirms its influence on cellular metabolism. Previous studies have shown that YAP/TAZ promotes fatty acid and triglyceride synthesis by upregulating FAS (11, 96), ACL (11), ACC (11, 96), and SCD-1 (11, 96) through upregulated SREBP1. In addition, YAP/TAZ reportedly upregulated 30-hydroxymethyl glutaryl coenzyme A reductase (96) in cultured C57BL/6 mouse hepatocytes, by upregulating SREBP2, which in turn increased cellular cholesterol synthesis. *In vitro* HCC experiments revealed that YAP/TAZ promoted cancer cell proliferation by increasing lipid formation (11).

The effect of ECM stiffness on cellular lipid metabolism is through AMPK, which results in reduced anabolic lipid metabolism and enhancement of fatty acid *β*-oxidation to generate ATP and meet cellular energy needs. Functionally, AMPK induces translocation of the fatty acid transporter CD36 to the cell membrane (85, 97, 98) and increases fatty acids ingestion. In addition, it phosphorylates and inactivates ACC (85, 99–101), blocking the extension of fatty acid chains and stimulating fatty acid oxidation. Apart from these, AMPK phosphorylates and inactivates 30-hydroxymethyl glutaryl coenzyme A reductase (85, 102), thereby reducing cholesterol synthesis and energy expenditure.

Furthermore, ECM stiffness regulates cellular metabolism by modulating the actin cytoskeleton, by activating Rho/ROCK signaling, which also plays a role in the regulation of lipid metabolism. The actin cytoskeleton regulates the location of LPL on the cell surface, and its activity by regulating transport of LPL vesicles within the cell (103, 104). In addition, it has been implicated in regulation of SREBP processing in the Golgi apparatus, hence influencing lipid biosynthesis (27, 105). Previous studies have also shown that ECM stiffness also activates PTEN *via* the Rho/ROCK signaling pathway, thereby regulating cholesterol metabolism (106). On the other hand, TXNIP inhibits lipogenesis (45, 107), although in cardiomyocyte-specific TXNIP knockout mice, low levels of SREBP2 expression were recorded in cardiomyocytes following TXNIP knockdown. In fact, TXNIP deficiency also led to myocardial beta-oxidation (108).

Taken together, these findings indicate that ECM stiffness acts *via* different pathways to upregulate SREBP activity, with its downstream lipid metabolic enzymes. In addition, it up-regulates activity of LDLR, CD36 and LPL on the cell membrane to participate in lipid metabolism, thus affecting cell proliferation, growth and reducing cell apoptosis.

## ECM Stiffness and Amino Acid Metabolism

**Figure 3:**
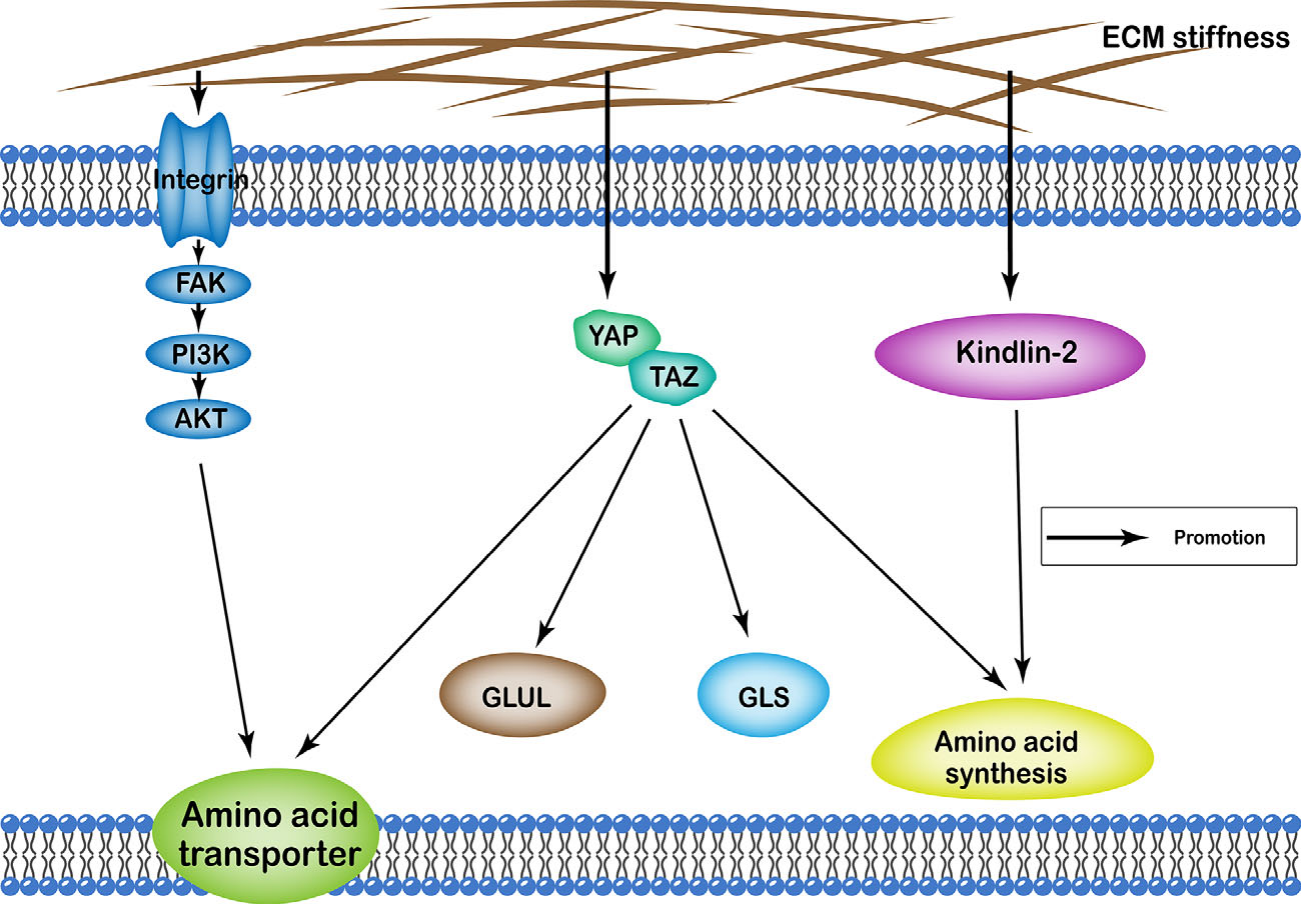
Profiles of pathways through which extracellular matrix stiffness affects amino acid metabolism. Extracellular matrix stiffness affects amino acid metabolism in the following three pathways: (i) YAP/TAZ pathway; (ii) kindlin-2 pathway; and (iii) integrin-FAK-PI3K-Akt pathway. The effects of extracellular matrix stiffness on cellular amino acid metabolism can ultimately be summarized as follows: (i) regulating the number of amino acid transport proteins in the cell membrane; (ii) regulating the quantity and activity of enzymes involved in the amino acid synthesis; (iii) regulation of glutamine catabolism; (iv) regulation of the synthesis of glutamine.

ECM stiffness regulates amino acid metabolism of cells through many different pathways, including the YAP/TAZ, and kindlin-2 pathways, as well as integrin-FAK-PI3K-Akt. Functionally, it affects cellular amino acid metabolism by regulating the number of proteins involved in amino acid transport in the cell membrane, the quantity and activity of enzymes involved in the amino acid synthesis, as well as glutamine catabolism and synthesis (**Figure 3**).

ECM stiffness has been shown to regulate cellular metabolism by upregulating YAP/TAZ, a key player in amino acid metabolism. It also regulates enzymes involved in amino acid synthesis, including phosphoserine aminotransferase 1 (PSAT1) (109–111), phosphoserine phosphatase (PSPH) (109, 111), and phosphoglycerate dehydrogenase (111). These enzymes play a key role in synthesis of serine and serine hydroxymethyltransferase 2 (SHMT2) (109), which are involved in the synthesis of glycine and glutamic-oxaloacetic transaminase (110) that in turn catalyze aspartate production. Previous studies have also shown that matrix stiffness can upregulate enzymes involved in the synthesis of amino acids by regulating the YAP/TAZ pathway. For example, *in vitro* knockout of a gene upstream of YAP/TAZ or YAP/TAZ gene downregulated YAP/TAZ and PSAT1, PSPH, and SHMT2, inhibited serine/glycine production repressed proliferation of prostate cancer cells (109). Conversely, downregulating YAP/TAZ and downstream PSAT1, PSPH, and SHMT2 genes in xenograft tumors of nude mice resulted in decreased tumor volume and weight (109).

ECM stiffness has been implicated in glutamine catabolism, through direct activation of YAP-TEAD-mediated glutaminase(GLS) transcription (30, 32, 112, 113). Glutamine catabolites participate in the TCA cycle, where they provide energy for cells. Apart from this, glutamine is a precursor for many non-essential amino acids, and also plays a key role in the synthesis of many amino acids. Bertero et al. found that the matrix stiffness-YAP/TAZ pathway in squamous cell carcinoma increased glutamate synthesis in cancer cells by upregulating GLS (32). The synthesized glutamate entered into cancer-associated fibroblasts to mediate synthesis of glutathione and balance the redox state and increase cell contractility. Consequently, the cancer-associated fibroblasts reportedly increased aspartate synthesis through upregulated GLS. The resultant aspartate entered cancer cells to participate in nucleotide synthesis and promote proliferation of cancer cells. siRNA-mediated inhibition of GLS resulted in reduced proliferation of cancer cells and inhibited their invasive ability. In murine breast cancer models, inhibiting ECM stiffness using BAPN or inhibiting YAP using verteporfin resulted in reduced proliferation of cancer cells, as well as tumor size, and number of lung metastases. In addition, the authors recorded prolonged survival following downregulation of GLS and activity (32). A TCGA-based analysis revealed an association between high levels of GLS mRNA in head and neck squamous cell carcinoma with overall poor prognosis of patients. Therefore, targeting these molecules for may be a feasible approach for treating the disease (32). In another study targeting pulmonary arterial hypertension, Bertero et al. reported reduced cell proliferation and migration in pulmonary arterial endothelial cells or pulmonary arterial smooth muscle cells following siRNA-mediated knockdown of GLS or YAP/TAZ genes (30). Previous studies, using mouse models, have also shown that pulmonary vascular matrix stiffness is involved in vascular cell proliferation through regulation of glutamine catabolism *via* YAP/TAZ. Inhibiting ECM stiffness using BAPN (LOX inhibitor) was found to downregulate YAP and GLS and reduce proliferation of endothelial and smooth muscle, atherosclerosis, and pulmonary hypertension (30). In addition, GLS inhibitors have also been found to inhibit growth and migration of myofibroblastic hepatic stellate cells (112), while glutaminase inhibition has been reported to reduce cell growth in breast cancer cells (113).

However, a metabolomic analysis, targeting a zebrafish model, reported that the YAP/TAZ pathway did not promote glutamine catabolism. Instead, the authors found elevated production of glutamine through upregulation and increased activity of glutamine synthetase (GLUL). Consequently, the increase in glutamine enhanced nucleotide biosynthesis and promoted liver growth (10). In zebrafish larvae, use of GLUL inhibitor methionine sulfoximine or knocking out GLUL significantly inhibited YAP-driven hepatomegaly, whereas the purine analog mycophenolic acid also suppressed the YAP-driven hepatomegaly. These findings demonstrate that YAP-mediated activation of GLUL increases glutamine levels, while promoting nucleotide biosynthesis is a significant factor in hepatomegaly or rapid cell proliferation in hepatocellular carcinoma (10).

ECM stiffness has also been implicated in amino acid transport, by upregulating YAP/TAZ. Specifically, YAP/TAZ upregulates the Asp/Glu transporter SLC1A3 (32), neutral amino acid transporter protein SLC1A5 (113), glutamine transporter SLC38A1 (114), leucine transporter SLC7A5 (114, 115), and SLC3A2 (115). Consequently, high SLC1A5 expression in breast cancer revealed a strong association with decreased overall breast cancer survival based on analysis of patient data from GEO database (113). On the other hand, Park et al. found that knocking down SLC38A1 or SLC7A5 significantly inhibited cell growth in HCC cells (114). However, cell growth was restored following exogenous introduction of SLC38A1 and SLC7A5 into YAP/TAZ-depleted cells *via* expression vectors. Moreover, knocking down SLC38A1 or SLC7A5 has been shown to significantly reduce tumor weight and growth in xenotransplantation models of subcutaneous and orthotopic HCC in nude mice. These results affirm the key role played by YAP/TAZ in growth and progression of HCC cells, through activation of SLC38A1 and SLC7A5. Taken together, these studies indicate that ECM stiffness plays a key role in synthesis and transport of amino acid, as well as synthesis and decomposition of glutamine by regulating YAP/TAZ activity. These processes are key in regulating growth, proliferation, invasiveness, and metastasis of cancer cells.

Previous studies have also described ECM stiffness’ critical role in tumorigenesis, survival, proliferation, and apoptosis. Generally, this is through the regulation of proline metabolism by kindlin-2, one of the molecules that regulate cellular and extracellular matrix adhesion. This molecule is also present in the mitochondria, where it forms complexes with PYCR1 (116, 117), a key enzyme that regulates proline synthesis. ECM stiffness has been shown to promote entrance of kindlin-2 into the mitochondria where it interacts with PYCR1, thus promoting proline synthesis (116). *In vitro* and *in vivo* experiments, targeting lung adenocarcinoma, have demonstrated that knocking down of the kindlin-2 gene increased ROS and apoptosis, but reduced the number of cells, as well as the percentage of Ki67-positive cells by reducing proline synthesis. This in turn affected lung carcinogenesis and reduced lung cancer mortality of mice (116).

Furthermore, ECM stiffness has been shown to enhance integrin signal and stimulate FAK activation, thereby activating PI3K/Akt signaling. Activated Akt signals play a key role in amino acid metabolism, and also increase abundance of the SLC6A19 protein, an amino acid transporter in the cytomembrane (118), as well as the 4F2hc (also known as the CD98) (95), which are key promoters of cellular uptake of amino acids.

The different pathways of ECM stiffness that regulate metabolism have mutual promotion or restriction. For example, studies have shown that GSK3 is inactivated under Akt regulation (119–121). Thus, ECM stiffness may participate in cellular metabolism by regulating GSK3 activity through Akt. In addition, TXNIP is regulated by Akt (44) and AMPK (43), although it is not clear whether ECM stiffness regulates cell metabolism by TXNIP through Akt and AMPK. Furthermore, ECM stiffness-mediated activation of Kindlin-2 has been associated with activation of integrin (122), with kindlin-2 shown to regulate YAP/TAZ signals at both transcriptional and protein levels (123). Therefore, it is possible that ECM stiffness regulates metabolism *via* the integrin-kindlin-2-YAP/TAZ signaling pathway. In summary, it is clear that ECM stiffness-mediated regulation of metabolism may vary across different types of cells.

## Conclusion

ECM stiffness plays a key role in the regulation of many aspects of cell activities that require metabolic energy supply, including survival, growth and development, proliferation, apoptosis, tumor development, migration and metastasis (124). In this review, we have described several mechanisms through which ECM stiffness regulates metabolism, and outlined their impact on cellular life activities and tumors. Summarily, ECM stiffness affects cell behavior by regulating tumor metabolism. This may explain why therapies targeting ECM of tumors have been in the focus of numerous researches over recent years. While these may provide effective solutions for controlling tumors, there is need to address various associated limitations to guarantee precision therapy targeting matrix stiffness and its regulated signaling pathways. For example, further research models are needed to unravel the interrelationship between tumor development and changes in matrix stiffness, since the two are mutually influenced. In addition, since the use of a single target molecule for therapy has proven to be less effective than the regulation of signaling pathways, focus needs to shift to targeting these pathways, owing to mutual regulation among them. Furthermore, there is need to address the challenge of model establishment and ensure they mimic the dynamics of matrix stiffness in disease scenario. This is because the existing models have posed difficulty in representing the heterogeneity of stiffness within tumors.

## Author Contributions

The manuscript was designed by HG, FT, and HP. The manuscript and figures were prepared by HG, MT, and QP. Further edits and revision were made by FT and HP. All authors contributed to the article and approved the submitted version.

## Funding

This study was supported by the Nature Scientific Foundation of China (grant no. 81702956), the Strategy-Oriented Special Project of Central South University in China (grant no. ZLXD2017003), the Natural Science Foundation of Hunan Province (grant nos. 2020JJ4903 and 2020JJ5920), and the colorectal cancer medical seed research fund project named “Effect and mechanism of YAP1 on EGFR resistance in K-ras wild-type metastatic colorectal cancer” from the Beijing Bethune Public Welfare Foundation.

## Conflict of Interest

The authors declare that the research was conducted in the absence of any commercial or financial relationships that could be construed as a potential conflict of interest.

## Abbreviations

ACC, acetyl-CoA carboxylase; ACL, ATP-citrate lyase; ECM, extracellular matrix; FAS, fatty acid synthase; GLS, glutaminase; GLUL, glutamine synthetase; GSK3, glycogen synthase kinase-3; G6Pase, glucose-6-phosphatase; HA, hyaluronic acid; HCC, hepatocellular carcinoma; HK2, hexokinase 2; LDHA, lactate dehydrogenase A; LDLR, low-density lipoprotein receptor; LPL, lipoprotein lipase; MPA, mycophenolic acid; MSO, methionine sulfoximine; PDAC, pancreatic ductal adenocarcinoma; PEPCK, phosphoenolpyruvate carboxykinase; PFK, phosphofructo kinase; PFKFB3, 6-phosphofructo-2-kinase/fructose-2,6-biphosphatase 3; PHGDH, phosphoglycerate dehydrogenase; PKM2, pyruvate kinase M2; PSAT1, phosphoserine aminotransferase 1; PSPH, phosphoserine phosphatase; SCD-1, Stearoyl-CoA desaturase 1; SHMT2, serine hydroxymethyltransferase 2; SREBP, sterol regulatory element-binding protein; TRIM21, E3 ubiquitin ligase tripartite motif (TRIM)-containing protein 21; TXNIP, thioredoxin-interacting protein.
